# Effectiveness of Preterm Infant Oral Motor Intervention (PIOMI) on oral feeding readiness among preterm infants: a quasi-experimental study

**DOI:** 10.1186/s12887-026-06781-y

**Published:** 2026-03-28

**Authors:** Amrita A. Sivasanker, Pity Koul, SreeRaja Kumar, Rajeev Kumar Thapar

**Affiliations:** 1 Sharda School of Nursing Science and Research, Sharda University, Uttar Pradesh Greater Noida, India; 2 Sharda School of Nursing Science and Research, Sharda University, Uttar Pradesh Greater Noida, India; 3 Department of Pediatrics, Sharda Hospital, Greater Noida, Uttar Pradesh India

**Keywords:** Infant, Premature, Oral stimulation, Development care, NICU

## Abstract

**Background:**

Preterm infants often experience feeding issues due to neuromuscular immaturity and underdeveloped oral motor skills leading to delayed oral feeding readiness and prolonged hospitalization. Preterm infant oral motor intervention (PIOMI) has been progressed as non-invasive strategy to enhance oral feeding readiness among preterm infants.

**Methods:**

A quasi-experimental research approach with non-randomized pretest post-test control group design was adopted. The study was carried out in a NICU of Tertiary care hospital in North India between February to October, 2024. A total of 130 Preterm infants (65 in each group) fulfilling inclusion criteria were assigned to an intervention group receiving oral stimulation twice daily for 7 days for 5 min and control group received routine care only. Baseline oral motor development was assessed and post-test was done on 1st, 4th and 7th days using preterm oral feeding readiness assessment scale (POFRAS). Data was analyzed using linear mixed-effects models (LMM) to evaluate the changes in oral motor development over time between groups.

**Results:**

Oral motor development scores were found to be comparable at baseline between intervention and control group (*p* > 0.05). The linear mixed- effects model showed a significant effect of time (*p* < 0.001) and a significant group X time interaction (*p* < 0.001), indicating greater improvement in oral feeding readiness among infants receiving PIOMI compared with only routine care. No significant association was found between demographic variables and oral motor development scores.

**Conclusions:**

The Preterm Infant Oral Motor Intervention significantly improved oral motor development among preterm infants. PIOMI is feasible nurse-led intervention that support oral feeding readiness among preterm infants.

**Trial registration:**

Clinical Trial Registry of India (CTRI/2023/11/060267). Registered on: 28/11/2023.

**Supplementary Information:**

The online version contains supplementary material available at 10.1186/s12887-026-06781-y.

## Introduction

Preterm infants often experience feeding difficulties due to neuromuscular immaturity and underdeveloped oral motor skills, which can delay the transition from tube to independent oral feeding and extend hospitalization. Delayed oral motor development is associated with prolonged dependency on gavage feeding, increased risk of growth failure, and caregiver burden in neonatal intensive care units (NICUs) where preterm infants are commonly managed [1]. Efficient oral motor coordination, encompassing sucking, swallowing, and breathing, is crucial for safe and effective oral feeding and is a key determinant of neonatal outcomes [2].

In NICU settings, prolonged dependency on enteral feeding as well as exposure to highly medicalized environment further hinders oral sensory input thus delaying the acquisition of oral motor skills especially in preterm infants [3]. These challenges highlight the need for developmentally supportive intervention that promotes oral maturation while ensuring safety and feasibility in routine clinical practice.

Oral motor interventions, including structured stimulation of Paediatric oral structures, are designed to enhance neuromotor coordination and sensory integration, facilitating earlier and more effective feeding in this high-risk population. Recent evidence suggests that oral motor stimulation protocols such as the Preterm Infant Oral Motor Intervention (PIOMI) can improve feeding performance and accelerate the transition to full oral feeding among preterm infants compared with routine care [4]. Systematic reviews and meta-analyses indicate that oral motor interventions, including nonnutritive sucking and oral sensory stimulation, may contribute to improved feeding readiness and reduced time to achieve oral feeding, although the certainty of evidence is variable [5, 6].

The preterm infant oral motor intervention (PIOMI) is an oral stimulation intervention that is focused on strengthening oral feeding skills especially in preterm babies. This intervention mainly focuses on chin, cheeks, lips, tongue, gums and palate in and around the mouth as preterm infants have restriction in stretching mouth as well as minimal tolerance of outside stress. As per literature, the preterm infants who receives oral stimulation at 29 weeks of postmenstrual age (PMA) significantly shows improvement in maturation, oral motor function, oral feeding skills and coordination while sucking [7, 8].

Interventions targeting oral motor development have also been associated with improvements in oral motor function and feeding skills when administered by healthcare professionals or caregivers in NICU settings [9]. However, variations in intervention protocols, outcome measures, and study designs have limited the generalizability of findings. In particular, there remains a need for focused evaluation of structured oral motor interventions on longitudinal changes in oral motor development scores, rather than solely time-to-feed outcomes.

Despite growing evidence supporting Preterm infant oral motor intervention (PIOMI) most of the studies only focuses on length of hospital stay, full oral feeding time and other feeding milestones. There remains a research gaps examining the longitudinal effects of structured oral stimulation on overall oral motor development across multiple post intervention time points [10]. Continuous evaluation of oral motor development may provide deeper insight into neuromuscular maturation and underlying feeding readiness mechanisms in preterm infants.

Given the clinical importance of early oral motor maturation for feeding progression and overall neonatal health, this study aimed to evaluate the effectiveness of a structured oral motor intervention on oral motor development among preterm infants admitted to the NICU, compared with routine hospital care.

## Methodology

### Study design

In this study, a quasi-experimental, non randomized pretest- posttest control group research design was employed to assess the effectiveness of oral motor stimulation on oral feeding readiness among preterm infants. Participants were assigned to intervention and control groups using a non-random sequential allocation approach. Preterm infants fulfilling eligibility criteria were first enrolled in the intervention group and subsequently in control group until the required sample size for both groups was achieved.

The baseline assessment (pretest) was conducted for preterm infants fulfilling the inclusion criteria in both intervention group and control group using socio-demographic proforma and standardized preterm oral feeding assessment readiness scale (POFRAS). After the baseline assessment intervention group received preterm infant oral motor intervention (PIOMI) stimulation in addition to routine NICU care, whereas the control group only received routine care. Post-test was done on Day 1^st^, Day 4^th^ and Day 7^th^ in both the groups.

This study is reported in accordance with the STROBE (Strengthening The Reporting of Observational studies in Epidemiology) guidelines. To minimize potential bias, standardized assessment tools were used for both groups and the same data collection procedures were followed throughout the study.

### Participants and setting

The sample size was calculated using power analysis based on findings from previous studies evaluating oral motor stimulation among preterm infants. Assuming a significance level of 0.05, statistical power of 80%, and an expected moderate effect size, the minimum required sample size was estimated to be 120 participants. To account for potential attrition, a total of 130 preterm infants (65 in each group) were enrolled in the study. This study was conducted on preterm infants admitted in Neonatal Intensive Care Unit of Sharda Hospital. The participants were preterm infants admitted to NICU during the study period.

### Inclusion criteria

Preterm infants included in the study were:

- ∙ Alert and awake during the time of assessment.
- ∙ Gestational age of 28–34 weeks.
- ∙ Atleast 72 h old.
- ∙ APGAR scoring more than 6.
- ∙ Clinical stable and tolerating enteral feeding.
- ∙ Admitted in NICU for less than 7 days.

The preterm infant oral motor intervention (PIOMI) was initiated only when the preterm infants were clinically stable. The intervention was started after at least 72 h of birth, once infants demonstrated physiological stability and tolerance to enteral feeding. Criteria for initiating oral stimulation included stable heart rate and oxygen saturation, absence of significant apnea or bradycardia episodes, and the ability to tolerate gavage feeding. Only preterm infants who were clinically stable and not on respiratory support at the time of intervention were included Infants receiving mechanical ventilation or continuous positive airway pressure (CPAP) having serious medical or surgical complications are excluded from the study.

### Preterm Infant Oral Motor Intervention (PIOMI)

The preterm infant oral motor intervention (PIOMI) was originally developed by Brenda Lessen in 2010 as a structured oral motor protocol to enhance oral feeding among preterm infants [11]. It involved five- minute structured oral motor stimulation technique designed to enhance oral feeding readiness and coordination.

This intervention can be effectively administered by trained nursing professional or even parents. The intervention comprises of structured oral massage targeting the chin, cheeks, lips, tongue, gums and palate followed by non nutritive sucking. Unlike the traditional oral motor massages, PIOMI emphasis on functional pressure and movement patterns to elicit oral motor responses.

The intervention was performed by first researcher who completed formal PIOMI training and received a certificate from the developer. For the intervention group, PIOMI was done for 5 min twice daily (morning & evening) in a day, beginning 30 min before the scheduled feeding time in addition to routine NICU care. The intervention continued for seven consecutive days.

Whereas, the preterm infants in control group received only routine NICU care. The post-test was done for both the groups.

### Data collection tools

Total two instruments were used in this study:

- ∙ Socio- demographic proforma: A structured socio-demographic proforma was used to collect the Gestational age of baby at birth, Gender of baby, Mode of delivery, Weight of baby, APGAR score at 1 min and 5 min.
- ∙ The Preterm Oral Feeding Readiness Assessment Scale (POFRAS) was developed and standardized by Dr. Fujinga et al., to assess the oral feeding readiness among preterm infants [12]. Written permission was obtained from developer to use this tool in the present study. It consisted of five categories including 18 items in total related with: (1) Corrected age, (2) State of behavioral organization (state of consciousness, global posture, general tone), (3) Oral posture (lip posture, tongue posture), (4) Oral reflexes (seeking, sucking, biting, gagging reflexes) and (5) non-nutritive sucking ( movement of fill, grooving of the tongue, movement of jaw, suction force, suctions per pause, suction rhythm maintenance by pause, maintaining alertness, signs of stress). These items were assessed using a 3- point scale where No response was denoted as 0, Weak or Slow response was denoted as 1 and Strong response was denoted as 2. The minimum scoring of tool was 28 whereas, maximum score was 36.
- ➢ The POFRAS is classified into following categories with scores:
  - *< 2*8points = No Oral Feeding,
  - 28–30 points= Initial Breast Feeding.
  - *≥* 30 points = Complete Breast Feeding.

In the present study reliability was checked again to assess the feasibility of the tool in Indian context and found to be 0.93 indicating high internal consistency and reliability.

### Data collection procedure

Preterm babies were enrolled from 1^st^ February 2024 to 31^st^ October 2024. A total of 130 preterm infants (65 in experimental and 65 in control group) were included in the study using total enumeration sampling technique. Sample size was calculated using power analysis and review of literature.

The prior permission for the study was obtained from institutional authorities. The objectives and procedure of the study were explained to staff nurses on duty. Rapport was established with mothers as well as primary care giver before the enrollment. Eligible Preterm infants were recruited in intervention group first and then in control group until required sample size was achieved.

Baseline assessment (pretest) was conducted for both groups using the socio-demographic proforma and POFRAS scale. The physiological stability of the preterm infants was assessed using standard NICU monitoring equipment including calibrated digital thermometer for temperature measurement, pulse oximeters for monitoring oxygen saturation and cardiac monitor for heart rate and respiratory rate assessment. Following the baseline assessment, the intervention protocol was implemented in the intervention group, and follow-up assessments were conducted on Day 1, Day 4, and Day 7 for both groups.

POFRAS assessments were performed by an independent assessor who was blinded to the group allocation to minimize assessment bias. Standardized assessment procedures were used throughout the study to ensure consistency and reduce measurement bias.

### Statistical analysis

Data was analyzed using IBM SPSS Statistics version 22. Descriptive statistics (frequency, percentage, mean and standard deviation) were used to summarize the data distribution. Normality of the data was tested using the Shapiro-Wilk test.

Independent t-test was used to compare POFRAS scores between the intervention and control group at each assessment points. To evaluate changes in oral motor development over time and difference between the intervention and control groups, a liner mixed effects model (LMM) was used. In the model intervention and control was treated as a between subject factor and time (pre-test, post- test I, post-test II and post-test III) was treated as within subject repeated factor. Participant ID was included as a random effect to account for within subjects correlations across repeated measurements.

The group × time interaction was examined to determine whether the trajectory of change in oral motor development differed between the intervention and control groups over the study period.

Additionally, the Chi-square test was used to examine the association between selected demographic variables and oral motor development scores. A *p*-value < 0.05 was considered statistically significant.

### Ethical approval and consideration

This study received ethical approval from Institutional Ethics Committee (IEC) of School of Medical Sciences and Research, Sharda University, Greater Noida (U.P) Ref. No. SU/SMS&R/76-A/2023/169. The study was conducted in accordance with ethical principles of declaration of Helinski. Informed consent was obtained from mothers who were clearly informed of their right to withdraw from the study at any time for any reason without penalty. The trial was registered under Clinical Trial Registry of India with reference number CTRI/2023/11/060267.

## Results

A total of 130 preterm infants (65 in each intervention and control groups) were enrolled in this study. Baseline demographic and clinical characteristics were comparable between the intervention and control groups with no statistically significant differences observed (*p* > 0.05), indicating homogeneity between the groups at baseline (Table 1).

**Table 1:** Baseline clinical characteristics of preterm infants in intervention and control group. *N* = 130 (*n*1 = 65, *n*2 = 65)

Variable	Intervention group Mean ± SD	Control group Mean ± SD	t	*P* value
Gestational age at birth (weeks)	31.2 ± 1.7	31.2 ± 1.8	0.46	0.65
Birth weight (kg)	1.79 ± 0.30	1.84 ± 0.39	0.72	0.47
Age at intervention initiation (days)	3.8 ± 0.9	3.9 ± 1.0	0.59	0.55
Age at assessment in days (post- test I)	4.8 ± 0.9	4.9 ± 1.0	0.57	0.57
Age at assessment in days (post-test II)	7.8 ± 0.9	7.9 ± 1.0	0.59	0.55
Age at assessment in days (post-test III)	10.8 ± 0.9	10.8 ± 1.0	0.00	1.00

The baseline clinical characteristics of preterm infants were comparable between the intervention and control groups. No statistically significant differences were observed in gestational age at birth, birth weight, age at intervention initiation, or age at assessment points (*p* > 0.05), indicating that both groups were similar at baseline.

### Changes in oral motor development scores across time

The mean oral motor development scores (POFRAS) of preterm infants at baseline and subsequent post intervention time points are represented in Table 2. At baseline the mean POFRAS scores in intervention group (26.33 ± 5.64) were comparable with that of the control group (25.06 ± 5.47), indicating no significant difference between the groups before the intervention.

After implementing the intervention, there was significant increase in POFRAS scores in intervention group across post test assessments. However, the control group shows only minimal changes.

**Table 2:** Comparison of POFRAS scores between intervention and control groups across different time points. *N* = 130 (*n*1 = 65, *n*2 = 65)

Time points	Intervention Mean ± SD	Control Mean ± SD	t test	*p* value
Pre-test	26.33 ± 5.64	25.06 ± 5.47	1.30	0.84
Day 1 (Post-test I)	26.35 ± 5.16	26.53 ± 5.50	0.19	0.84
Day 4 (Post – test II)	29.55 ± 3.70	26.76 ± 5.08	3.56	0.001*
Day 7 (Post -test III)	31.49 ± 3.28	26.64 ± 4.68	4.0	0.001*

*Statistically significant at *p* < 0.05

The baseline POFRAS scores were comparable between the intervention and control groups (*p* = 0.84). No significant difference was observed on Day 1 after the intervention (*p* = 0.84). However, the intervention group demonstrated significantly higher POFRAS scores on Day 4 (*p* < 0.001) and Day 7 (*p* = 0.001) compared with the control group. These findings indicate that preterm infants receiving the PIOMI intervention showed greater improvement in oral feeding readiness than those receiving routine care alone.

Between groups comparison of POFRAS scores at each time point were performed using independent t-test to evaluate the effect of intervention while accounting for natural maturation in preterm infants.

### Linear Mixed-Effects Model Analysis

A linear mixed effect model analysis (LMM) was conducted to examine the effectiveness of intervention on oral motor development over the time points (Table 2). The model includes groups i.e. intervention and control as between subject factor and time i.e. pre test, post test I, post test II, post test III as repeated within subject factor with participant ID specified as a random effect.

The analysis revealed a significant main effect of time on POFRAS scores (F(3,128) = 33.397, *p* < 0.001) indicating that oral motor development scores have changed significantly across the study period. The main effect of groups was not statistically significant (F(1,128) = 1.869, *p* = 0.174), highlighting that there was no overall difference between the intervention and control group when averaged across all time period.

Moreover, a significant group X time interaction effect was observed (F(3,128) = 7.410, *p* < 0.001), indicating that the trajectory of oral motor development differed significantly between the intervention and control groups. Specifically, preterm infant who received the preterm infant oral motor intervention (PIOMI) showed significantly greater improvement in oral motor development scores over time when compared with preterm infants receiving only routine hospital care.

These findings further suggests that the observed improvements in oral motor development among preterm infants were attributable to intervention rather than normal maturation alone (Table 3).

**Table 3:** Linear Mixed- Model examining the effect of group and time in POFRAS scores. *N* = 130 (*n*1 = 65, *n*2 = 65)

Fixed effect	Numerator df	Denominator df	F value	*p* value
Group	1	128	1.869	0.174
Time	3	128	33.397	< 0.001*
Group x time	3	128	7.410	< 0.001*

*Significant at *p* < 0.05

**Fig. 1:**
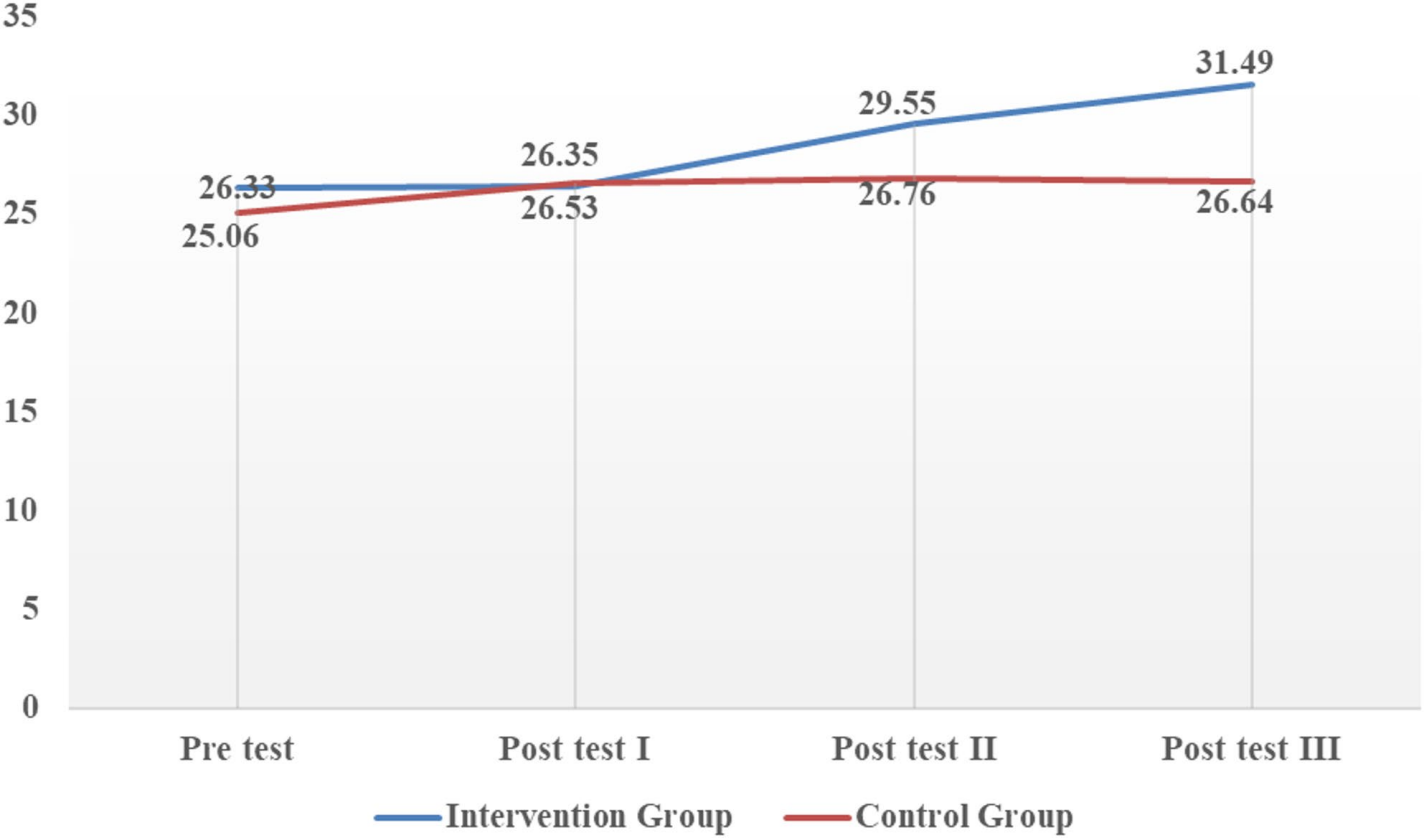
Changes in mean oral motor development (POFRAS) scores among preterm infants in intervention and control groups across different study time points

Figure 1. Shows the changes in mean oral motor development among preterm infants from pretest to post test III at different points. At baseline both groups demonstrated comparable oral motor development scores. There was no change observed at post test I. Whereas, post test II onwards shows progressive increase in intervention group while remaining largely unchanged in control group.

In the present study, no statistically significant association between selected background and clinical variables including gestational age, gender, mode of delivery, birth weight and APGAR scores at 1 and 5 min—and oral motor development outcomes. These findings indicate that improvements in oral motor development were attributable to intervention and not influenced by baseline demographic characteristics. However, it is also possible that the sample size of the present study may not have been sufficient to detect smaller associations between demographic variables and feeding readiness outcomes. Future studies with larger sample sizes may help further explore the potential moderating effects of these clinical characteristics.

## Discussion

The present study examined the effectiveness of the Preterm Infant Oral Motor Intervention (PIOMI) on oral motor development among preterm infants admitted to the NICU. At baseline, oral motor development scores were comparable between the intervention and control groups, indicating that both groups were similar prior to the implementation of the intervention. This baseline equivalence suggests that any subsequent improvements observed during the study period are likely attributable to the intervention rather than pre-existing differences between the groups.

Following the implementation of PIOMI, preterm infants in the intervention group demonstrated a progressive improvement in oral motor development scores across successive assessment points. In contrast, the control group showed only minimal changes over time. The linear mixed-effects model analysis revealed a significant effect of time as well as a significant group × time interaction, indicating that the trajectory of oral motor development differed between the intervention and control groups. These findings suggest that repeated exposure to structured oral stimulation contributed to enhanced oral motor development among preterm infants.

The progressive increase in oral motor scores observed in the intervention group highlights the importance of repeated and structured stimulation for promoting neuromuscular maturation of oral structures in preterm infants. Oral stimulation techniques such as PIOMI are designed to facilitate coordination of sucking, swallowing, and breathing, which are essential for successful oral feeding. The lack of immediate improvement at the early post-intervention stage observed in this study may indicate that oral motor development requires continuous stimulation over time rather than producing instantaneous effects.

The findings of the present study are consistent with previous quasi-experimental studies that have reported improvements in oral feeding readiness and oral motor skills following oral stimulation interventions in preterm infants [13]. Similarly, randomized controlled trials evaluating oral motor stimulation programs have demonstrated enhanced feeding performance and faster progression to independent oral feeding among preterm infants receiving structured stimulation compared with those receiving routine care [14].

The significant time effect observed in the current study suggests that oral motor development improved progressively throughout the intervention period. However, the presence of a significant group × time interaction further indicates that the improvement observed in the intervention group was greater than what could be expected from natural maturation alone. These results support earlier experimental studies reporting that oral motor stimulation interventions can accelerate the development of feeding skills and facilitate earlier attainment of independent oral feeding among preterm infants [15, 16].

In addition, the present study found no statistically significant association between selected demographic and clinical characteristics, including gestational age, gender, mode of delivery, and APGAR scores, and oral motor development outcomes. This finding suggests that the effectiveness of the intervention was not influenced by these background characteristics. Similar findings have been reported in previous clinical studies, which also indicated that demographic variables did not significantly affect feeding outcomes following oral motor stimulation interventions [17].

Overall, the findings of this study support the effectiveness of structured oral motor stimulation in enhancing oral motor development among preterm infants. The observed improvements across repeated assessments emphasize the importance of incorporating developmentally supportive interventions such as PIOMI into routine neonatal care practices.

### Limitations and future research

This study has limitations. The findings of study cannot be generalized due to small sample size. The study was conducted in single NICU that limits its applicability into different setting. The study period was limited to 7 days only which restricted the observation of gradual changes in developmental assessment of preterm infants.

Further longitudinal research should be done to develop a institutional protocol that can be implemented to check feasibility of oral stimulation among preterm infants as a routine procedure. Educational programmes need to designed and organized to sensitize healthcare workers as well as parents for participation in oral stimulation to promote developmental neonatal outcomes. Similar study can be replicated on larger sample in multiple settings.

Further longitudinal research studies can be conducted to explore the long-term effectiveness of preterm infant oral motor intervention (PIOMI) in terms of oral motor skills, feeding milestones as well as cognitive abilities in preterm infants. A qualitative study can be conducted to explore the satisfaction level of mothers and healthcare workers after performing oral motor stimulation among preterm infants.

Another limitation of the present study is that outcomes were limited to oral feeding readiness as assessed by POFRAS scale. Functional feeding outcomes like quality of sucking, feeding efficiency, time of achieve full oral feeding or milk intake were not evaluated. So further studies can be done to include these functional outcomes as well to provide more comprehensive assessment of oral motor intervention among preterm infants.

## Conclusion

This study demonstrated that the Preterm Infant Oral Motor Intervention significantly improved oral motor development among preterm infants when compared with routine care alone as reflected by higher POFRAS scores over time. These findings suggest that structured oral motor stimulation may support the development of oral feeding readiness in preterm infants.

## Supplementary Information

Supplementary Material 1.

## Data Availability

The datasets used or analyzed during the current study are available in anonymized form from the corresponding author on reasonable request.

## Abbreviations

PIOMI: Preterm Infant Oral Motor Intervention
POFRAS: Preterm Oral Feeding Readiness Assessment Scale
NICU: Neonatal Intensive Care Unit
ANOVA: Analysis of Variance
SPO2: Saturation of oxygen
