# Supplementary material for: Effectiveness of Preterm Infant Oral Motor Intervention (PIOMI) on oral feeding readiness among preterm infants: a quasi-experimental study
Source: BMC Pediatr. 2026 Mar 28;26:417. doi: 10.1186/s12887-026-06781-y (PMC13151247; doi:10.1186/s12887-026-06781-y)
Supplement: Supplementary file 1 — Supplementary Material 1. [file 12887_2026_6781_MOESM1_ESM.docx]

# STROBE Checklist

Strengthening the Reporting of Observational Studies in Epidemiology

1. Title and abstract: Indicate the study’s design and provide a balanced summary. 1

2. Background/rationale: Explain scientific background and rationale. 2

3. Objectives: State specific objectives and hypotheses. 3

4. Study design: Present key elements early. 5

5. Setting: Describe setting, locations, and relevant dates. 5

6. Participants: Eligibility criteria and selection methods. 5

7. Variables: Clearly define outcomes and exposures. 6

8. Data sources/measurement: Describe assessment methods. 5

9. Bias: Describe efforts to address potential bias. 5

10. Study size: Explain how the study size was determined. 5

11. Quantitative variables: Explain handling of variables. 6

12. Statistical methods: Describe methods used. 7

13. Participants flow: Numbers at each stage. 6

14. Descriptive data: Characteristics of participants. 6

15. Outcome data: Report outcomes. 7

16. Main results: Unadjusted estimates and precision. 7

17. Other analyses: Subgroups or interactions. 7,8,9

18. Key results: Summarize key findings. 8

19. Limitations: Discuss limitations. 11

20. Interpretation: Cautious interpretation. 11

21. Generalisability: External validity. 11

22. Funding: Source and role of funders.12
